# An integrative approach for analyzing hundreds of neurons in task performing mice using wide-field calcium imaging

**DOI:** 10.1038/srep20986

**Published:** 2016-02-08

**Authors:** Ali I. Mohammed, Howard J. Gritton, Hua-an Tseng, Mark E. Bucklin, Zhaojie Yao, Xue Han

**Affiliations:** 1 Boston University, Department of Biomedical Engineering, Boston, MA 02215.

## Abstract

Advances in neurotechnology have been integral to the investigation of neural circuit function in systems neuroscience. Recent improvements in high performance fluorescent sensors and scientific CMOS cameras enables optical imaging of neural networks at a much larger scale. While exciting technical advances demonstrate the potential of this technique, further improvement in data acquisition and analysis, especially those that allow effective processing of increasingly larger datasets, would greatly promote the application of optical imaging in systems neuroscience. Here we demonstrate the ability of wide-field imaging to capture the concurrent dynamic activity from hundreds to thousands of neurons over millimeters of brain tissue in behaving mice. This system allows the visualization of morphological details at a higher spatial resolution than has been previously achieved using similar functional imaging modalities. To analyze the expansive data sets, we developed software to facilitate rapid downstream data processing. Using this system, we show that a large fraction of anatomically distinct hippocampal neurons respond to discrete environmental stimuli associated with classical conditioning, and that the observed temporal dynamics of transient calcium signals are sufficient for exploring certain spatiotemporal features of large neural networks.

Technology development plays an important role in systems neuroscience and has greatly advanced our understanding of how individual neurons encode, or represent, environmental stimuli or cognitive processes. For example, the development of intracellular and extracellular electrophysiology techniques, capable of measuring electrical signals from individual neurons or ensemble populations with a high spatiotemporal resolution, has led to important insights into the temporal dynamics of neural signals related to behaviors^1^,^2^,^3^,^4^. EEG, MEG, fMRI, and PET, although limited in their spatiotemporal precision, allow non-invasive access to populations of cells in animal models and humans. In addition, optical techniques, relying on the unique cell and tissue penetrating properties of light, have also been extensively explored for monitoring many individual cells or cell compartments. Most recently, new generations of high performance voltage^5^ and calcium sensors^6^,^7^,^8^ have proven effective as temporally precise indicators of neural activity in behaving animals. In particular, the latest generation of genetically encoded GCaMP6 calcium sensors exhibit a high level of sensitivity, capable of resolving single action potentials^9^,^10^.

Optical imaging for neuroscience applications has traditionally involved either wide-field imaging or two-photon imaging, each with distinct advantages and disadvantages^11^. Two-photon microscopy, with its superb spatial resolution and deep tissue penetrating ability, has been the preeminent choice in recent years^9^,^12^,^13^,^14^,^15^. Recent studies using two-photon imaging have revealed important insights into how individual cell types contribute to the coding of specific stimuli within neural networks^13^,^16^. Because two-photon microscopy employs a scanning mechanism, the signal to noise ratio is primarily influenced by the time spent on imaging each point, and the spatial resolution is primarily determined by the number of points scanned for each image. As a result, the size of the imaging field is inversely correlated with the overall temporal resolution when the signal-to-noise ratio is kept constant. To achieve a relatively high signal-to-noise ratio, conventional two-photon calcium imaging is often performed on small brain areas or across a sparse network of cells, to maintain temporal fidelity.

Wide-field imaging of neural tissue – either through a microscope or similarly constructed macroscope has proved a useful tool in neuroscience labs for several decades. This approach was first employed to characterize vascular architecture and measure functional indicators of neural activity such as hemodynamic changes in brain tissue^17^. This technique’s popularity has seen a renaissance recently due to its simple instrumentation, relatively low cost, and the revolutionary improvement in the fidelity and stability of neural signal indicators. This combined with the synergistic increase in the sensitivity and dynamic range of optoelectronic sensors used for fluorescence detection has made this an increasingly viable tool for neuroscience researchers. New innovations, including the ability to perform wide-field calcium imaging in freely moving animals, through miniaturized microendoscopes^8^, promises to increase applicability for the types of experiments that can be performed using this technique. Although wide-field imaging lacks the spatial resolution to resolve the finest subcellular structures or to measure deeper tissue, it is capable of resolving clear neurites and somatic features for reliable spike detection^18^. Wide-field microscopy, unlike two-photon imaging, does not rely on scanning features, so it can be used to sample across a large field without having to sacrifice temporal resolution. Additionally, fluorophores may be less bleached using wide-field imaging compared to two photon imaging^19^,^20^, which makes it ideally suited for extended imaging sessions that may be particularly desirable for recording neural networks during some behaviors (*e.g.,* repeated trial learning paradigms). Thus, wide-field imaging offers unique advantages if the main scientific objective is to simultaneously record a large number of neurons in the brain with high temporal fidelity.

An emerging technical challenge that parallels advances in imaging large brain areas with high spatiotemporal resolution is the capture and processing of correspondingly large datasets. In this report we demonstrate the application of wide-field epifluorescence imaging using GCaMP6 and a scientific CMOS camera to record a large number of neurons in behaving animals at high spatiotemporal resolution. We also illustrate the challenges inherent to imaging and analyzing data from behaving animals and describe our approach to meet these challenges.

## Results

### Wide-field imaging of hippocampal CA1 neurons in awake behaving mice

**Figure 1:**
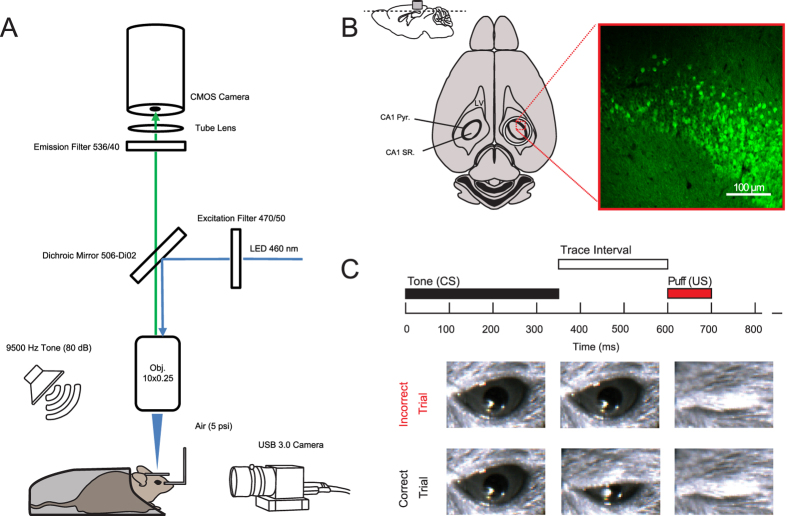
Experimental setup and behavioral design. (**A**) Diagram of image acquisition system and behavioral apparatus. Ca^2+^ signals were captured using a CMOS camera and illumination was achieved using a 460 nm LED. Animals were positioned via a head holder under a 10X objective lens. Air puffs were delivered via a cannula directed at the right eye and a USB 3.0 camera was used to monitor eyelid position at 20 Hz. Auditory cues were delivered at 80 dB from a speaker positioned behind the animal. (**B**) Anatomical depiction of cannula placement and imaging plane. A representative confocal image from the animal analyzed in Figs 2, 3, 4, 5. Cannula is to scale: note that dorsal CA1 pyramidal cell layer below the cannula (CA1 pyr: stratum pyramidale; SR, stratum radiatum; LV, lateral ventricle). (**C**) Trace eye-blink paradigm. A 350 ms duration, 9500 Hz pure tone served as the conditioned stimulus (CS). The CS was followed by a 250 ms trace interval, which was followed by a 5 psi, 100 ms long, air puff to the eye that served as the unconditioned stimulus (US). Eyelid displacement was analyzed offline at the conclusion of the recording.

To demonstrate the use of wide-field microscopy across large brain areas at high spatial and temporal resolution we constructed a conventional epifluorescence microscope for recording in awake head fixed mice. The microscope consists of a 10X objective lens for increased imaging area, a high-intensity LED that can be precisely controlled via TTL pulses, a filter set appropriate for imaging GCaMP6 fluorescence, and a scientific CMOS camera capable of imaging large areas at high speed (Fig. 1A). In order to achieve high image quality with good signal-to-noise ratio, we imaged the hippocampal CA1 region; the CA1 region consists of a thin and densely packed pyramidal cell layer nested between two thick and sparsely populated layers (Fig. 1B). This unique anatomical organization of CA1 makes it ideal for wide-field imaging, because there is limited fluorescent signal adjacent to the imaging plane to contaminate the signals from the pyramidal cell bodies.

Mice were surgically injected with AAV-synapsin-GCaMP6f virus into the CA1 pyramidal cell layer, and then chronically implanted with an imaging window for optical recording (Fig. 1B). To illustrate the potential of using this system, we utilized the fastest GCaMP6, GCaMP6f, in reporting neural activity changes during behavior. We trained mice on a simple trace conditioning behavioral task that is known to depend on the hippocampus^21^,^22^,^23^,^24^, and involve CA1 neurons^25^,^26^,^27^,^28^. In trace conditioning, animals come to associate two stimuli that are otherwise unrelated. In this case, the conditioned stimulus, a 350 ms long tone, precedes the unconditioned stimulus, a gentle air puff to one eye, with a 250 ms interval (the trace interval) (Fig. 1C). Each training session consisted of 40 tone-puff trials with a 31–36 second randomized inter-trial interval (ITI). Performance is quantified via anticipatory eye lid movement that occurs in response to tone, but precedes air puff (Fig. 1C). Trace eye blink conditioning is a well-established behavioral paradigm, where mice generally learn the association over 1–3 behavioral training sessions^22^,^24^,^29^,^30^.

**Figure 2:**
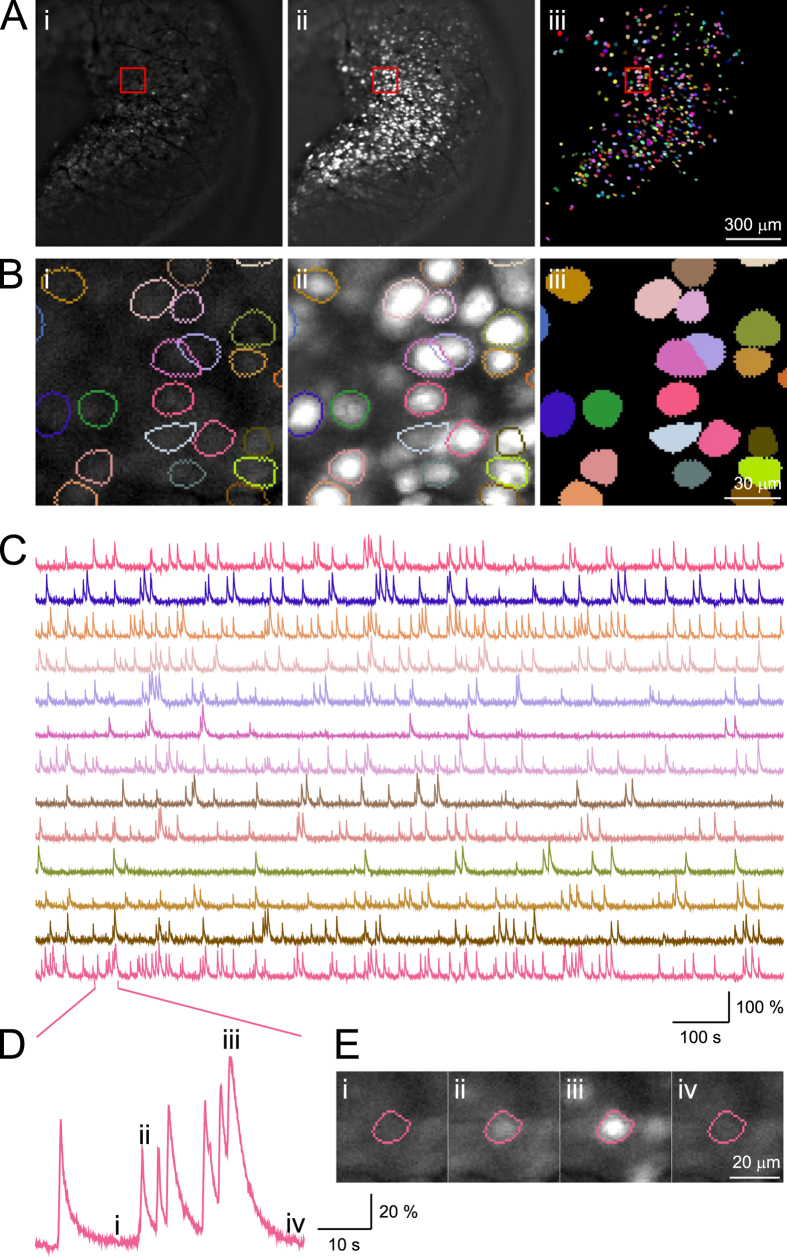
Imaging and identification of individual CA1 neurons. (**Ai**) A representative image frame from a standard imaging session (imaging field is 1343 × 1343 µm^2^). (**Aii**) Projection of maximum fluorescence intensity value across all frames. (**Aiii**) Color plot of all 422 semi-automatically identified neurons superimposed onto the imaging field of view. (**B**) Zoom in illustration from the area highlighted in the red box from (**A**) (the field is 131.2 × 131.2 µm^2^). (**C**) Fluorescence traces extracted for neurons shown in (**B**), with the color for each trace matching those presented in (**B**). Single dimensional fluorescence amplitude was independently scaled for each neuron. (**D**,**E**) An example neuron that exhibits highly dynamic features. (**Di**,**iv**) indicate periods with little activity, and (**Dii**,**iii**) indicate periods with higher levels of activity.

Fluorescence imaging was performed at 20 Hz with an image resolution of 1024 × 1024 pixels, while an animal was performing trace conditioning training (Supplemental Videos 1, 2 and 3). Although higher resolution and higher sampling rates are achievable (30–100 Hz depending on hardware configuration), this intermediate value allowed us to capture large areas while maintaining high fidelity. The camera was coupled to a 10X objective lens and thus each pixel corresponds to 1.312 × 1.312 μm^2^, which yields an imaging field of view of 1.343 × 1.343 mm^2^ (Fig. 2Ai,ii). Because CA1 pyramidal cells exhibit a diameter of approximately 15 μm, each cell is represented by several tens to hundreds of pixels, providing a high degree of morphological detail where large dendrites are often clearly observable, especially when they are adjacent to cell bodies (Supplemental Videos 2 and 3). Imaging data were acquired at 16 bits/pixel, which results in about 50 GB of imaging data in a typical 25 minutes recording session. The recording duration was chosen to match other eye-blink studies in terms of the number of trials presented and the duration of the inter-trial interval^22^,^24^,^29^,^30^. Data acquisition was performed with the commercial software package HCImageLive running on a multicore computer. We streamed data from the camera directly to RAM to ensure precisely timed acquisition and to avoid potential frame dropping associated with write delays and buffer overflow. At the end of a recording session, imaging data were transferred from RAM to the hard drive for long-term storage and processing. Although, imaging data can be streamed directly from camera to high speed solid state drives during acquisition, we found that direct streaming in this manner resulted in a small number of missing frames within a behavior session. Behavioral stimuli and image acquisition were triggered by TTL pulses that were controlled via customized MATLAB functions, and recorded for offline validation. Imaging data were stored as multi-page tagged image file format (mpTIFF) and processed offline.

### Image processing for motion correction and region of interest (ROI) identification

We first performed a series of image pre-processing steps to attenuate motion artifact that is associated with inherent physiological processes, such as respiration, changes in blood flow, or skeletal muscle movement that directly influences the position of the brain. Because the brain is surrounded by cerebral spinal fluid within the skull, any flexing in skeletal plates or jaw movements promote brain displacement in head fixed mice. Motion induced changes in the XY plane can largely be corrected during the image registration process, but changes in depth (Z) or non-rigid deformations in the imaging plane are more troublesome and cannot be easily compensated for.

The motion correction process begins with contrast enhancement to correct for any non-uniformity in illumination of the imaging plane to enhance features for better calculation of correlation in later steps. The motion correction process utilizes phase correlation to measure displacement between the current frame and a reference image consisting of all previously corrected frames averaged together. The frame is then shifted in the XY plane to offset the displacement. The displacement between images determined during motion correction provides a metric of the magnitude of motion within a recording session. We found that the magnitude of displacement for each imaging session remained relatively constant across animals, with an average of 0.96 ± 1.32 µm/frame (mean ± standard deviation, over the 3 imaging sessions analyzed across all 3 mice). We note that z-direction motion cannot be compensated during image processing and that future improvements in hardware instrumentation will be critical to correct this type of motion. In the following analysis, we present detailed characterization from a representative mouse dataset (mouse 26) with an overall displacement of 1.18 ± 1.65 µm/frame (mean ± standard deviation across frames). Population data for motion displacement, behavioral performance, and task relevant ROIs for all 3 mice recorded and analyzed are summarized in Tables 1, 2, 3.

Prior to motion correction, we observed small rhythmic motion artifacts that were periodic in nature and occurred with a frequency of ~2–3 Hz, consistent with the respiration rate for quietly awake mice (Supplemental Fig. 1A,B). We also observed larger and infrequent motion artifacts associated with skeletal movements, such as grooming or posture shifts, which produced biphasic responses containing both positive and negative changes. These artifacts were stereotypical, and were restricted to a few frames, which made them easily dissociable from calcium transients that exhibit an exponential decay and last tens of frames or longer. Our rigid image registration algorithm greatly reduced the majority of both types of motion (Fig. S1). In the instances where motion artifacts remained, it is likely attributable to z-plane motion or sub-pixel non-rigid deformations where it has been reported that fluctuations of up to 2 μm can occur in head fixed animals^31^. Movement of this magnitude in two-photon imaging can alter signal dramatically as the scanning plane limits the spatial resolution to a few microns. Subtle z-plane movements, however, have a much smaller impact in a wide-field imaging preparation because the focal plane spans tens of microns.

After motion correction, regions of interest (ROIs) were isolated according to the spatial distribution of the fluorescent signals at each pixel. We first selected pixels with relatively high intensity within each frame, and then clustered them into single-frame ROIs using proximity between activated pixels. Single-frame ROIs were then grouped and merged based on spatially overlapping presentations across frames, and restricted by CA1 neuron morphology. Upon completion of automatic analysis, our software identified 1086 ROIs in the example dataset from mouse 26. This algorithm does not utilize traditional principle component analysis (PCA). Instead it applies fluorescence intensity thresholds for pixel selection and limits the spatial extent of pixel comparisons, thus requiring significantly fewer computational resources and processing data at much faster speeds. Moreover, the computational resources used by this algorithm scales linearly with image resolution, a feature critically needed for analyzing increasingly large datasets in any reasonable period of time. This is in contrast to traditional algorithms like PCA that scale exponentially with image size. Finally, we calculated the signal-to-noise ratio for each of the identified ROI’s to determine signal quality. We found that the mean signal-to-noise ratio of all ROIs exceeded 6 in all animals (12.92 ± 0.44, mean ± standard error of mean; see Table 2).

Next, we manually inspected each ROI from the example dataset to determine how the automatic algorithm performed in identifying individual ROIs (Fig. 2B). We confirmed that ROI identification was successful when labelling was relatively sparse. However, upon visual inspection, we found that at areas where labelling was dense, it is in general difficult to distinguish cells that present greater spatial overlaps, especially when these overlapping cells also have significant temporally coincident fluorescence signals. We utilized the default set of parameters in our automatic ROI identification algorithm, with the goal of maximizing the identified number of truly distinct cells, even if they overlap spatially. In order to obtain clearly separable signals from distinct ROIs, we manually selected 422 ROIs that exhibited clear single neuron morphology, as well as defined spatial segregation (Fig. 2Aiii). This stringent manual selection process eliminated ROIs that may faithfully represent single neuron activities, but allowed us to avoid repeating or over-representing pixels originating from neurons that share the same anatomical location. A better dissociation would likely be achieved through sparser labeling, as well as better use of temporally segregated fluorescent signals.

Upon completion of semi-automated neuron identification, we extracted the averaged fluorescent intensity of the 422 ROIs for subsequent analysis (Fig. 2C). Upon closer examination of the 422 ROIs, we found that these ROIs exhibit fluorescence changes that matched the expected GCaMP6f temporal dynamics associated with neural activity (Fig. 2D). Different ROIs exhibit vastly different features, with some sparsely active and others highly dynamic (Fig. 2C). For some ROIs, there are clear examples of summation of fluorescence intensity over brief time intervals, likely corresponding to sustained neuron activity over a short period (Fig. 2D,E). The highly interspersed temporal patterns of Ca^2+^ responses are consistent with that reported in electrophysiology^32^, and imaging studies of CA1 pyramidal cells^8^,^33^. Based on this evidence, we concluded that these selected ROIs represent individual CA1 neurons.

### Calcium dynamics of hippocampal neurons during trace conditioning behavior

**Figure 3:**
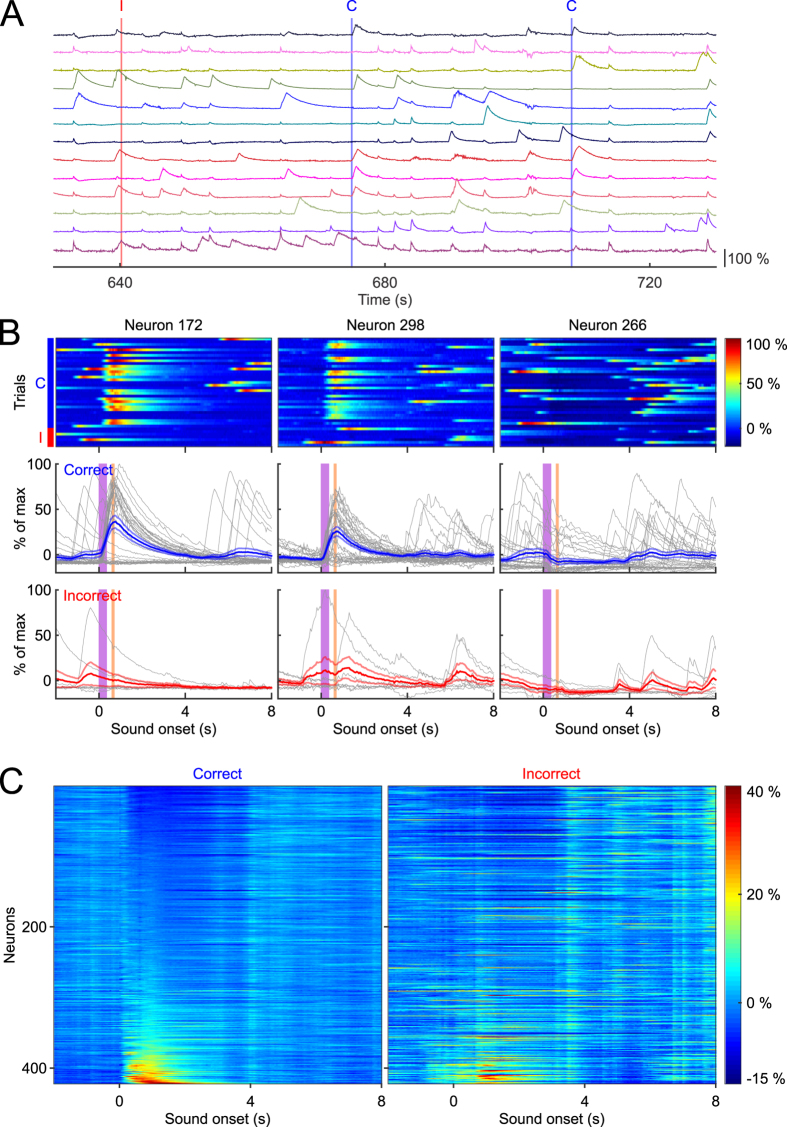
Tone evoked responses sorted by response magnitude. (**A**) Individual traces from 13 representative neurons from a continuous 3 trial window of time. The red vertical demarcation indicates an incorrect trial (labelled with “I” at the top), and the blue lines indicate correct trials (labelled with “C” at the top). (**B**) Responses of three individual neurons plotted for all 40 trials. Top: plots of Ca^2+^ response magnitude sorted by correct trials (33 trials on the top, indicated by the blue bar to the left) and incorrect trials (7 trials at the bottom, indicated by the red bar to the left). Bottom: individual responses sorted by trial outcome, with each trial response plotted in gray, and averaged response ± SEM plotted in blue or red depending on trial outcome. (**C**) Mean response amplitude of all neurons sorted by least responsive (top) to most responsive (bottom), during 0–2 seconds of tone onset, separated by trial outcome.

We characterized task related neural activity in the identified CA1 neurons by aligning Ca^2+^ responses to tone onset, and then sorting by trial outcome (Fig. 3). Trial outcome was assessed by monitoring eyelid position via a USB camera, and calculating eyelid displacement in the interval from tone onset until puff onset. Correct trials were defined as those with significant eyelid displacement relative to baseline, and prior to the air puff, whereas incorrect trails were defined as lack of significant eyelid displacement. For the dataset quantified here, behavioral performance was 82%, consistent with asymptotic performance of 50–90% reported in other trace eye-blink studies^22^,^29^,^30^. Behavioral performance data for all animals are reported in Table 2.

We found that many neurons showed robust changes upon tone or puff presentation, either showing an increase in fluorescence intensity or a cessation of activity that lasted several seconds. Several neurons showed Ca^2+^ transients correlated with trial outcome in that they were much more likely to respond to the tone on correct trials as opposed to incorrect trials (Fig. 3B, Neurons 172 and 298). Interestingly, some neurons were less likely to exhibit Ca^2+^ transients immediately after tone onset, or a cessation of activity, and thus showed an overall reduction in their trial averaged Ca^2+^ responses (Fig. 3B, Neuron 266). This reduction lasted several seconds, which cannot be explained by the brief fluorescence changes associated with motion mediated artifacts that typically last tens to hundreds of milliseconds (Fig. S1). It was also apparent that many neurons exhibited differential responses depending on whether the animal correctly performed the trial. These findings parallel prior electrophysiology studies demonstrating that the hippocampus codes for relevant task stimuli, and that some neurons exhibit differential neural activity depending on trial outcome^26^.

To estimate task involvement from the entire population of simultaneously recorded neurons in this animal, we sorted the 422 neurons based on their mean responses during the 2 second window following tone onset for correct trials (Fig. 3C left, 33 correct trials), and for incorrect trials (Fig. 3C right, 7 incorrect trials). Upon further analysis, we found that 41% of these neurons showed significant changes within 2 seconds of tone onset on correct trials, with 81 neurons (18%) exhibiting increases in Ca^2+^ responses, and 102 (23%) showing a reduction. We note that because of the limited number of trials used here, it is possible that more neurons may have been modulated but failed to reach statistical significance. In general, our estimation of 41% is consistent with findings from prior electrophysiology studies^23^,^26^,^27^,^34^,^35^. Together, these results demonstrate that Ca^2+^ changes in CA1 can be matched to relevant task stimuli in a classical eye-blink conditioning task, and highlight that GCaMP6f is sensitive enough to represent neuronal dynamics that reflects comparable results from electrophysiology studies.

To be certain that motion-induced artifact was not contributing to our estimations of individual ROI’s responses, we carefully compared changes in fluorescence signal intensity from periods with high image registration index for each ROI. We found that the largest motion events often produced small deflections with brief onsets and offsets, which are small in amplitude and much faster when compared to the observed Ca^2+^ signals associated with cell firing (see Fig. S1A,B). In addition, non-motion corrected data yielded a similar number of positively and negatively modulated neurons (Table 1), suggesting that even uncorrected motion artifact has a negligible impact on observed Ca^2+^ dynamics in our recording sessions. Interestingly, we did find some neurons’ had Ca^2+^ responses correlate with motion onset throughout the entire behavioral session, exhibiting prolonged temporal profiles typical of cell activation (Fig. S1B), suggesting that some hippocampal neurons reflect motor related activity.

We then analyzed the responses from the automatically detected 1086 ROIs, using the same categorization criteria. 590 ROIs (53%) showed significant changes in the 2 second window following tone onset, with 216 (19%) exhibiting an increase in Ca^2+^ responses, and 374 (34%) showing a reduction. These ratios are remarkably similar to that obtained from the smaller dataset that were manually selected, suggesting that our automatic ROI detection algorithms achieved reasonable performance. We then applied the same algorithm to classify the neuronal responses from the datasets of all animals recorded and found that in every animal, both positively and negatively modulated subsets of neurons could be identified (see Table 1). While it remains unclear how different parameters would impact ROI identification accuracy; additional signal processing methods, such as the ones that evaluate multi-dimensional spatiotemporal interactions^36^, will likely improve the performance of automatic ROI identification algorithms. Such automated algorithms will make it feasible to process increasingly large datasets associated with behavioral experiments that often involve multiple sessions from many animals.

### Temporal resolution of GCaMP6f fluorescence signals in representing neural network dynamics

**Figure 4:**
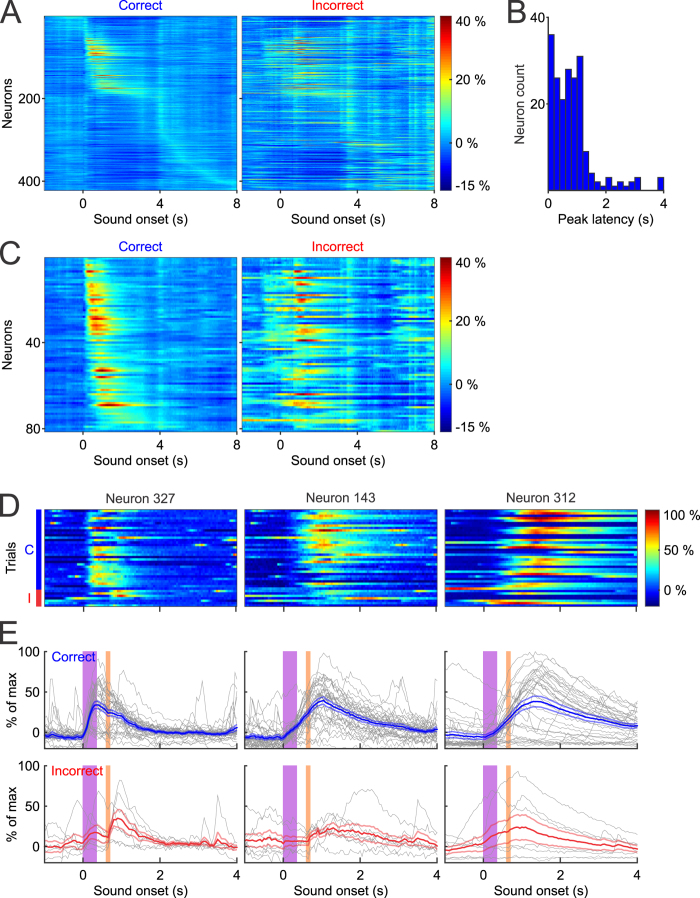
Tone evoked responses sorted by latency to peak amplitude. (**A**) Neurons sorted based on latency following tone presentation over an 8 second window. (**B**) Histogram distribution of the latency for neurons that reached peak activity within 4 seconds of tone onset. (**C**) Statistically positively modulated neurons (n = 81) sorted by peak latency by trial outcome. (**D, E**) Three representative neurons that exhibit variability in response kinetics, defined as the rate of fluorescence increase after tone onset. Individual trial responses were plotted in gray, and mean responses ± SEM were plotted in blue or red, depending on trial outcome.

Neural network computation by necessity must happen on a rapid time scale, and therefore, maintaining the fidelity of neural interactions is highly relevant in systems neuroscience. Electrophysiology has a unique advantage in temporal precision, capable of resolving the timing of action potentials with sub millisecond precision. To evaluate the temporal resolution of individual Ca^2+^ transients in representing neural dynamics of behavior, we analyzed the time when calcium responses reached the peak after tone onset in the example dataset. We sorted the latency to peak amplitude for all neurons from tone onset until 8 seconds later (Fig. 4A), and found that a large portion of the population (184 out of 422) reached their peak within 2 seconds of tone onset (Fig. 4B). Of these 184 neurons, 83 reached their peak within 600 ms of tone onset, prior to the puff, suggesting that they are likely responsive to the tone. The remaining 101 neurons peaked between 600 ms and 2 seconds after tone onset, which could represent either sustained activity during the trace interval or a response to the air puff.

We also noticed that many neurons (238 out of 422 ROIs) that did not show a peak response in the 2 second window analyzed, reaching their peak intensity within the several seconds that followed. It is intriguing that these neurons showed delayed peaks that extend into the ITI period, which may reflect the general involvement of CA1 in the maintenance or storage of recent events. Although we cannot specifically pinpoint the functional significance of these delayed signals, it suggests that task relevant signals may exist in windows of time that have traditionally been deemed task inconsequential.

We then further analyzed the population of 81 neurons that were positively modulated, whose response dynamics can be easily characterized (Fig. 4C). These neurons exhibit distinct temporal patterns and reached their peak fluorescence at different time points after tone presentation during correct trials. Interesting, their activity patterns during incorrect trials are rather different, with many neurons showing robust responses to puff onset instead of tone onset. The differential response profiles between correct and incorrect trials further confirm the task dependent properties of these neurons.

In addition to the difference in the latency to peak amplitude, we also observed distinct kinetic differences in the rate of fluorescence changes. Some neurons showed rapid rises in calcium and reached peak amplitude shortly after tone onset (Fig. 4D,E, ROI 327), while others rose more slowly and took longer to reach peak amplitude (Fig. 4D,E, ROI 143 and 312). These findings suggest that Ca^2+^ response profiles in the hippocampus can occur over wide time scales, consistent with a functional role in possibly binding distinct events during behavioral trace intervals^23^,^37^. Taken together, our results indicate that calcium imaging using GCaMP6f is sufficiently fast to discriminate neural responses to discrete behavioral elements of the trace eye-blink task.

### Anatomical clustering in CA1 hippocampal networks during trace conditioning

One of the benefits of using wide-field imaging is the ability to sample over very large areas at high temporal resolution. Using our particular experimental configuration, we were able to measure across 1.343 × 1.343 mm^2^, with a theoretical maximum spatial resolution of 1.312 × 1.312 µm^2^/pixel without considering light scattering in brain tissue. This distance is large enough to capture a majority of the dorsal CA1 region, allowing us to address previously intractable questions, such as the spatial and temporal topography of neurons over large areas. For example, electrophysiology experiments have suggested that CA1 neurons that respond to a given environmental stimulus are often largely heterogeneous and intermixed^32^,^38^,^39^; although, there may be some local organization over very small distances, due to the likelihood of receiving coincident inputs as in spatial hippocampal maps^40^.

**Figure 5:**
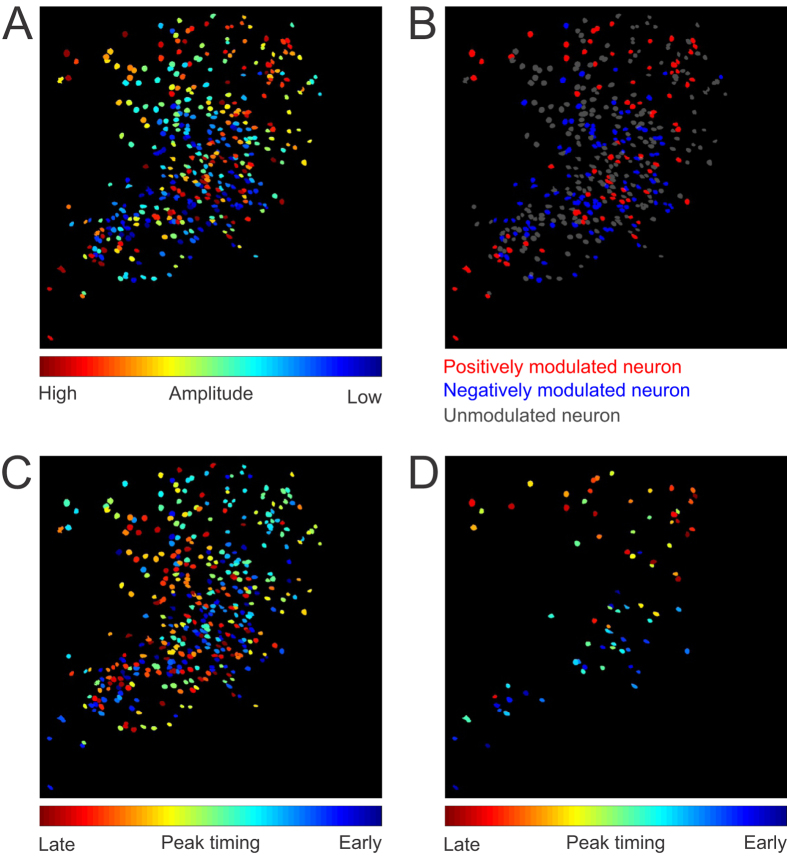
Spatial organization of task related CA1 neurons. (**A**) Spatial distribution of all 422 neurons sorted by averaged amplitude during the cue-response window (0–2 seconds after tone onset). Neurons in dark red demonstrate the highest peak amplitude, whereas neurons in dark blue represent biggest negative amplitudes during this window. (**B**) Neurons are colored according to their task relevance, with red being significantly positively modulated (n = 81 neurons), blue being significantly negatively modulated (n = 102 neurons), and gray being not modulated (n = 239 neurons). Note, some anatomical clustering of the same color labelled neurons within close proximity to one another. (**C**) Spatial distribution of all 422 neurons sorted by the latency to peak amplitude. Neurons in blue reached peak amplitude quickly, whereas neurons in red reached peak amplitude more slowly. (**D**) Spatial distribution of the 81 positively modulated neurons, sorted by the latency to peak amplitude.

In order to examine the spatial distribution of cells that are task relevant, we mapped responses of individual neurons based on their relative anatomical locations. We plotted the location of all 422 neurons from the example dataset based on their average amplitude during the cue-response window defined as the 2 seconds following tone onset (Fig. 5A). We did not identify any spatial patterns across the whole imaging field, but did notice that many neurons with similar responses tend to cluster together. For example, we saw neurons that were often in close proximity to other neurons with similar responses (shown as similar colors in Fig. 5A). We further color coded neurons that are either positively modulated (Fig. 5B, red), negatively modulated (blue), or unmodulated (gray). Interestingly, positively modulated neurons tended to be largely interspersed throughout the recording window (median distance between cells = 479.05 μm; Resampling test p = 0.002, Fig. 5B and Fig. S3A) when compared to the median distance between all cells resampled (402.23 ± 23.48 μm, mean of median ± standard deviation of median). We then looked to see if this phenomenon was conserved across all animals. We found that positively modulated ROIs were more sparsely distributed across all subjects (567.1 ± 107.6 μm; mean ± SD; F(1,2) = 26.88, p = 0.035) than non-modulated neurons (517.8 ± 96.5 μm; mean ± SD; F(1,2) = 0.01, p = 0.914) when compared to the population distribution. This finding reveals that one advantage of monitoring calcium dynamics over large areas is that that the simultaneous activity of functionally interconnected neurons spread over large distances may be sampled in the same imaging window. In addition, the sensitivity to both increases and decreases of activity could be used to map reciprocal relationships that may differ in polarity of the response based on functional connectivity or anatomical location.

In the example shown in Fig. 5B, it is also easily observable that some positively modulated neurons (red) are often in close proximity to other positively modulated neurons, and negatively modulated neurons tend to be next to other negatively modulated neurons, thus forming small red or blue clusters with each containing a few cells. In order to quantify this effect, we calculated the proximal distribution of neurons of each type (positive, negative, or non-modulated) within a 50 μm distance from the center of each ROI in all animals. We found that across all animals (see Table 3), positively modulated neurons tend to be adjacent to other positively modulated neurons as opposed to all other cell types (p = 0.003, z = 2.97, Mann-Whitney). Such correlated responses in small networks is consistent with previous observations during trace condition using two-photon imaging^28^, and future development in data analysis techniques will likely reveal important insights on the anatomical significance of different functional network activity patterns.

We also looked at whether neurons might cluster by the time course of their responses for all 422 neurons (Fig. 5C), and for the 81 positively modulated neurons (Fig. 5D). We found no topographical organization based on response latencies when neurons were compared to one another, consistent with what has been previously observed in CA1 cells during spatial navigation^32^. Interestingly, the small clusters of cells that share similarity in amplitude modulation are not observed in the latency maps, suggesting heterogeneously intermixed temporal patterns of task relevant CA1 neurons, even at small distances. Together, we found evidence for both spatial and non-spatial organizations across different spatial scales. While at very small scales, some neurons may form clusters according to their task related amplitude modulation, such clusters were not observed when timing was considered. Moreover, while no distinct clusters are observed across the large CA1 network, there may be enhanced segregation between positively modulated neurons.

## Discussion

In this report we have outlined a simple and economical wide-field microscopy optical imaging system that allows for recording the simultaneous activity of hundreds to thousands of neurons in task performing animals. We also demonstrate a software that processes large data sets and identifies the simultaneous activity of hundreds to thousands of neurons. This system is easy to implement and handles large datasets with relative ease. We also address several technical issues related to motion correction, ROI selection, and neuron identification. Although several solutions currently exist for the processing of imaging data as open-source code, no one utility processes full datasets from pre-processing to ROI identification. The software described here combines many of these existing algorithms and features into a single utility that can effectively handle large datasets quickly and effectively, with clear advantages in terms of output, processing time, application, cost, autonomy, and usability.

We used traditional image registration algorithms for motion correction that rely on nonlinear least-square optimization solutions that have been developed for functional imaging data which work well and are freely available^41^. For example, the NIH program ImageJ, and the associated plugin TurboReg works well for image registration on small datasets of <4 GB^18^,^42^,^43^. However, as data size increases, ImageJ requires increasing demands on user input and processing time, with large files >30 GB taking 4–8 times longer when compared to this software using the same computer. This includes the time spent to manually load files, initiate concatenation, and to start the registration process (see Table 4). Once finished, user input is also required to save the output files for further processing. Each of these steps is automated using the software provided here. Another advantage is that the software here is not limited by available desktop RAM. The use of ImageJ required available desktop RAM at 2.5X the original recording file, limiting the size of files that can be processed by standard computers. In addition to automating the registration process, we also automated ROI detection as an alternative to the commonly used method of defining ROIs manually. In order to do this we utilize a threshold based ROI selection algorithm. This method does not rely on principle component analysis (PCA) or independent component analysis (ICA), thereby requiring significantly less computational resources and time. Excellent software algorithms are available that apply PCA/ICA based algorithms^43^ and are well suited for ROI identification. However, we found that on our large datasets, computational time was prohibitive to daily data collection and analysis. Therefore we developed an algorithm that applies an adaptive threshold to normalized pixel intensity and groups neighboring thresholded pixels into single-frame ROIs based on proximity (see methods for details). Single frame ROIs were then clustered into multi-frame ROIs that were used to describe the spatial extent of each highlighted neuron. We found that this produced reliable ROI identification in approximately 1/12 of the time of PCA based strategies using the same analysis computer.

We applied this system to recordings from CA1 neurons in a well characterized hippocampal dependent task and confirmed that the calcium dynamics observed in a large fraction of these neurons are task related, consistent with prior electrophysiology studies^23^,^26^,^27^,^34^,^35^,^44^,^45^. Because of the ability to image a large portion of the CA1 region, we found that task related CA1 neurons showed little clustering across a large spatial and temporal scales, but tended to form small spatial clusters that were modulated in response amplitude but not response latencies. We also demonstrate that Ca^2+^ imaging provides sufficient temporal resolution to measure neural network dynamics associated with distinct phases of behavior.

Based on our characterization, we conclude that for a variety of tasks, wide field imaging offers the potential to answer questions that other techniques may not. Although GCaMP6f has an improved temporal response relative to other genetically encoded Ca^2+^ indicators, its temporal resolution remains limited to resolving signals separated by tens or hundreds of milliseconds. Electrophysiology has far better temporal resolution when it can be applied, but may not be suitable for all experimental conditions. Future advances are likely to alleviate some of these concerns as larger and less invasive electrical recording arrays are developed along with the technology to enhance sampling bandwidth. As of now though, if information from large populations or from specific cell types that can be genetically targeted is required, Ca^2+^ imaging provides a unique alternative. In addition, Ca^2+^ imaging offers the potential to capture more sparsely active neurons providing an opportunity to analyze additional network dynamics that might otherwise be missed.

We found that, although minor, motion artifacts are an omnipresent issue inherent to recordings made in awake behaving animals. It is likely that motion will remain an inherent problem in all imaging techniques, as many physiological processes introduce micro-movements that need to be compensated for. We observed small rhythmic motion, such as respiration and heart rate, that is virtually undetectable following motion registration. For larger motion artifact due to skeletal muscle movement, such as grooming or posture shifts, our motion correction algorithms largely attenuated this motion artifact. Any remaining artifact was easily identifiable as a very stereotypical change in the ROI response that was temporally brief showing maximum displacement correction within one to two frames, as shown in supplemental Fig. 1A. It is also important to note that response profiles related to movement differ dramatically in their signal intensity, temporal profile (fast-onset and fast off-set), and correlated profile across all ROI traces. It has been estimated that the false-positive rate associated with motion-related artifact is less than 2% when higher signal-to-noise ratios, across longer temporal profiles are considered (i.e., >1.5 seconds)^46^. Based on the significant differences in the temporal profile of motion artifact, and those Ca^2+^ transients observed during cell firing, it is unlikely that motion artifact would be a sizable contributing factor to the number of significantly identified task-related ROIs measured here. Despite the success of our algorithm, we do believe that imaging at a faster speed could further improve motion correction, because motion displacement between frames will be minimized. Additionally, the use of a second fluorescent label utilizing a Ca^2+^ insensitive fluorophore might offer better motion estimation, and serve as a more ideal template for ROI identification. Another advantage of a second label would be that it could provide a better estimation regarding the number of neurons in the recording field, regardless of neural activity.

Wide-field imaging can be easily applicable to different brain regions and can be used to target specific cell types through transgenic or viral based gene targeting strategies^47^,^48^. Wide-field imaging also provides a solution for long term tracking of neural signals over days or weeks offering insight into questions about how individual cell populations come to form ensembles and how such representations are stabilized over days and weeks^8^. While two-photon has been a prime choice for imaging neural activities because of its superb spatiotemporal resolution, wide-field imaging represents a complementary choice at lower cost for imaging a larger brain area at higher speed. Wide-field imaging also may be less sensitive to photobleaching^19^,^20^, allowing for much longer continuous recordings without the fear of a deleterious reduction in signal. Over the ~25 minute recording sessions analyzed in this manuscript, we found photobleaching accounted for ~6.7% reduction in signal intensity across all animals (6.70 ± 5.74%, mean ± SD).

An additional future challenge of recording from larger number of neurons with high spatial and temporal resolution will be downstream image processing and data analysis, particularly as better voltage and calcium indicators are developed with finer temporal profiles. Further improvement of signal processing algorithms as well as data acquisition procedures would allow better representation and segregation of optical signals from individual neurons in wide-field imaging. Considering the potential to record from thousands to tens of thousands of neurons is in the foreseeable future, it will be beneficial to optimize data processing and analysis algorithms^43^,^49^. It is also unclear how different signal processing methods might bias neuron selection or activity detection, underscoring the importance of contrasting sorting methodologies. Beyond identification, analysis of interactions amongst thousands of individual neurons across behavioral conditions is going to require novel tools that increase the ease of performing multi-dimensional analysis^36^. It is our view that the continued development of optical imaging methods in systems neuroscience fundamentally relies on a collective effort that furthers the development of analysis software tools available via open access.

## Methods

### Animal Surgery

All animal procedures were approved by the Boston University Institutional Animal Care and Use Committee, and the methods were carried out in accordance with the approved guidelines. Female C57BL/6 mice, 8–12 week old at the start of the experiments, were used in all studies (Taconic; Hudson, NY). Mice were first injected with AAV9-Syn-GCaMP6f.WPRE.SV40 virus obtained from the University of Pennsylvania Vector Core (titer ~6e12 GC/ml). 250 nL of virus was stereotaxically injected into the CA1 region (AP: –2 mm, ML: 1.4 mm, DV: –1.6 mm) using a 10 nL syringe (World Precision Instruments, Sarasota, FL) fitted with a 33 gauge needle (NF33BL; World Precision Instruments, Sarasota, FL), at a speed of 40 µl/min controlled via a microsyringe pump (UltraMicroPump3–4; World Precision Instruments, Sarasota, FL).

Upon complete recovery, animals were surgically implanted with custom imaging windows, that consisted of a stainless steel cannula (OD: 0.317 in., ID: 0.236 in., height 2 mm), adhered to a circular coverslip (size 0; OD: 3 mm) using a UV-curable optical adhesive (Norland Products). After careful aspiration of the overlying cortical tissue, using the corpus callosum as an anatomical guide, the imaging window was placed above the CA1 viral injection site. During the same surgery, a custom aluminum head-plate was attached to the skull anterior to the imaging cannula.

### Animal Behavior

#### Trial Configuration

Individual trials consisted of a 350 ms, 9500 Hz pure tone (CS), digitized at 100 kHz with a 5 ms cosine ramp, delivered at 80 dB SPL. The tone was followed by a 250 ms trace interval and then a 100 ms long 5psi air puff (US) controlled via a solenoid (Clippard EV-2, Cincinnati, OH) and delivered through a 0.5 mm cannula positioned 2–3 cm away from the right eye. Trials occurred randomly with an inter-trial interval of 31–36 seconds.

#### Animal training

Mice were trained to criterion on a conditioned trace eye-blink task. Training was modified slightly from other previously published paradigms^24^,^28^. Briefly, animals were allowed to completely recover from surgery (typically about 4 weeks) before being handled and habituated to the training apparatus for 3 consecutive days. During habituation and training, animals were head fixed to custom holder that consisted of a 34 mm diameter aluminum half-tube that supported the animal and allowed for attachment of the head-plate at the anterior end. Animals were covered on top by an elastic wrap that reduced upward movement out of the half-tube. Habituation occurred at the same time as subsequent training (4–8 hours after lights-on). Following the habituation period, animals began training on the conditioned eye-blink task. With the exception of the first day of training, mice were trained in two blocks of 40 trials each. On the first day of training animals received 20 sound alone trials to determine a baseline level of eye-blink response for the tone prior to any tone-puff pairing. Subsequent training (days 2–5) consisted of 2 blocks of tone-puff training. A single 40 trial block took approximately 25 minutes. Animals were then given a 10–15 minute rest period before being trained on the second block. Prior to the training session animals were positioned underneath the CMOS camera and had the air-puff tube and a USB3.0 camera oriented for air puff delivery and eye movement capture, respectively. A custom MATLAB script controlled the behavioral stimuli and image capture timing using TTLs delivered via a I/O interface (USB-6259; National Instruments, Austin, TX). Image capture for both cameras were time locked to each other and sampling occurred at 20 Hz. Exposure time was fixed at 35 ms.

Eyelid position was monitored using Point Grey FlyCapture 2 software and Flea3 USB3.0 camera (FL3-U3-13S2C-CS; Richmond, BC, Canada). Eyelid position was calculated by converting the area of the eye into a region of interest using ImageJ (NIH; http://imagej.nih.gov/ij/). The eye and surrounding area was illuminated using an IR lamp positioned approximately 0.5 meters away. Using this configuration, eye lid area can be recorded as an increase in reflection that occurs as the eyelid closes impinging on the defined eye ROI.

#### Behavioral Analysis

Correct trials were characterized as those with changes in reflection that surpassed a significance threshold defined by that trials baseline activity. The threshold interval consisted of the 600 ms period following CS onset but prior to air-puff delivery. The baseline reflection values were calculated from the 3 second interval just prior to sound onset and reflect the deviation of reflection values used to determine the threshold value that was applied during the response window using the following criterion:


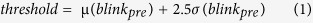


Trials were characterized then as correct or incorrect using this criterion and that standard was applied in separating ROIs based on behavioral outcome. A total of four animals underwent training with all four animals demonstrating a high level of conditioned responding (>50%) by the fourth day of training. One animal was excluded from analysis because of a high baseline startle response rate to sound only presentation prior to CS-US pairing.

### Microscope, camera, and hippocampal imaging

Image acquisition was performed via custom microscope equipped with a scientific CMOS (sCMOS) camera (ORCA-Flash4.0 LT Digital CMOS camera C11440-42U; Hamamatsu , Boston, MA). Fluorescence excitation is accomplished with a 5W LED (LZ1-00B200, 460 nm; LedEngin, San Jose CA). Image optics included a Leica N Plan 10 × 0.25 PH1 microscope objective lens, an excitation filter (HQ 470/50), dichroic mirror (FF506-Di02), emission filter (FF01-536/40), and a commercial SLR lens focused to infinity as the tube lens (Nikon Zoom-NIKKOR 80–200 mm f/4 AI-s). Hippocampal imaging data were collected and processed using a computer equipped with dual Intel Xeon processors, 128 GB RAM, and a GeForce GTX Titan video card. The use of the graphics card allowed for images to be processed offline by the GPU and therefore did not dependent on substantial amounts of available computer RAM. Images were captured as multi-page tagged image file format (mpTIFF) using the default image-capture software bundled with the purchase of a sCMOS camera from Hamamatsu (HC Image Live; Hamamatsu; Boston, MA).

### Photobleaching

Signal loss due to fluorophore bleaching was estimated by calculating the mean whole field florescence intensity reduction from the first until the final frame across the ~25 minute recording session.

### Image Processing

Image processing was performed offline using MATLAB software. The goal of this procedure was to reduce the raw image sequence to a collection of one-dimensional traces, where each trace indicates the fluorescence intensity of an individual neuron over time, and the collection approximates the distinct activity of each and every neuron in the imaging field of view. We implement the process in 3 distinct stages (Fig. S4) as described below.

#### Image Pre-processing: Contrast Enhancement and Motion Correction

Alignment of each frame in the image sequence with all other frames is essential to the methods we use in subsequent steps for identifying and tracking pixels over time. Thus, the goal of the first stage is to correct for any misalignment caused by movement of the brain tissue relative to the microscope and camera.

We preceded our main motion correction procedure with a contrast enhancement step that attempts to mitigate the effect of any non-uniform illumination of the brain tissue. Any illumination non-uniformity will be stationary in the camera’s field of view despite motion of the illuminated brain, which would hamper motion detection accuracy, or alternatively produce artifacts in accurately motion-corrected frames if left uncorrected. This enhancement is essentially a high-pass filtering operation, however the filtering must be performed in the log domain because of the multiplicative nature of light absorption. Additionally, artifacts often associated with digital high-pass filtering can be mitigated by equivalently computing a low-pass filtered component of each frame, then subtracting the low-pass component from the original image. This log-domain filtering operation is often described as “Homomorphic filtering” (see for an example; http://blogs.mathworks.com/steve/2013/06/25/homomorphic-filtering-part-1/).

Many algorithms for estimating and correcting image displacement exist and are well described in the medical imaging literature. We elected to use phase-correlation to estimate the induced motion in each frame, as we found this method to be highly stable, moderately accurate, and most importantly, fast, especially when implemented in the frequency domain and using a quality graphics card.

The operation estimates the mean translational displacement between two frames, one being the template or “fixed” frame, and the other being the uncorrected or “moving” frame. In the spatial domain this is accomplished by computing the normalized cross-correlation, which implies a 2-dimensional convolution of large matrices. The equivalent operation in the frequency domain is a simple scalar dot-product of the discrete Fourier transforms of each image normalized by the square of the template, followed by the inverse Fourier transform. The intermediate result is the cross-correlation (or phase-correlation) matrix, which should have a peak in its center for correctly aligned images, or a peak near the center, the offset of which indicates the mean offset between the two images. This peak can be found with subpixel precision by interpolation to give a more accurate alignment, although at some moderate expense in computation time.

For the template image we used a moving average of previously aligned frames when processing frames sequentially, which was averaged with a fixed mean of randomly sampled and sequentially aligned images from the entire set when processing files in parallel. The simplest way to perform this operation is to use the built-in MATLAB function *normxcorr2*, which makes optimization decisions based on image size and available hardware automatically. However, performance can be improved by tailoring the operation to your particular hardware and image size, i.e. using *fft2* and *ifft2* for large images and a good graphics card. Additional details of this procedure are well documented in the literature, as well as in the example functions provided at the following URL: http://www.bu.edu/hanlab/resources/. Shifting and interpolation are also covered in our MATLAB documentation and examples.

After aligning sequential image frames, we were able to estimate a baseline value and other statistics for each pixel (maximum, minimum, mean, standard-deviation, etc.), and use these statistics to reduce the bit-depth of frames being passed to the next stage. This was done purely to speed computation in subsequent steps, but won’t necessarily be helpful in all cases. For the results presented here, the motion-corrected images were saved as a new video file with 8 bit dynamic range, with the top 1% intensity saturated at 255 and the bottom 1% set at 0.

#### Region of Interest (ROI) detection

The ROI detection process used an adaptive threshold on the z-score of pixel intensity to reduce each frame to binary 1′s and 0′s (logical true or false). These binary frames were then processed using morphological operations to find and label connected components within each frame. For example, beginning with a z-score threshold of 1.5, all pixels that were more than 1.5 standard deviations above their mean were reduced to 1 (true), and all others reduced to 0 (false). Pixels reduced to 1 were often pixels overlying a cell that was significantly brighter during that frame due to activation of GCaMP. This initial threshold was adjusted up or down based on the number of non-zero pixels detected with each threshold. This was done to prevent spurious motion-induced shifts of the image frame from producing ROIs along high contrast borders. All morphological operations were performed using built-in MATLAB functions from the Image Processing Toolbox, which have fast parallel versions if the operation is run on a graphics card (e.g. *imclose, imopen*, etc.). Furthermore, the connected-component labeling and region formation operations were run using built-in MATLAB functions *bwconncomp*, and *regionprops*. Connected components were stored in a custom class and termed “single-frame ROIs,” and these were then passed to the 3^rd^ stage of processing, which merges them into a “multi-frame ROI” that represents the location and spatial distribution of each cell identified over the entire video.

#### Region of Interest (ROI) merging

The standard structure of region properties output by the MATLAB function *regionprops* (Area, BoundingBox, Centroid, etc.) are mimicked in a custom function called *RegionOfInterest*, where each field of the structure becomes a property of the custom class. We added additional properties for storing state information and data associated with each ROI, along with a number of methods for comparing, merging, manipulating, and visualizing the single-frame and multi-frame ROIs. The single-frame to multi-frame ROI merging procedure is essentially a clustering process that merges single-frame ROIs together using such criteria as the proximity of their centroids, as well as proximity of their bounding-box (upper-left and lower-right corners). Performing this operation quickly was highly dependent on pre-grouping ROIs based on centroid location in overlapping blocks of the image frame, as well as grouping by size. This enabled the clustering to be performed in parallel (across CPU cores) followed by a second iteration of clustering to deal with redundancy in overlapping regions.

Once ROIs are established, all video data is reloaded and passed to a method in the *RegionOfInterest* class that extracts the 1-dimensional trace for each ROI representing the fluorescence intensity in that region over time. The ROIs and their traces can then be quickly visualized using another method in the *RegionOfInterest* class, which relies on the *distinguishable_colors* function, available on the Mathworks File Exchange (http://www.mathworks.com/matlabcentral/fileexchange/29702-generate-maximally-perceptually-distinct-colors/all_files).

### ROI Trace Analysis

Signal-to-noise ratios were calculated for each ROI to estimate signal quality. The noise level was calculated by taking the pixel intensity of all pixels that were not registered to an ROI over time to calculate the average standard deviation of the noise. Then individual max ROI intensities were divided by the calculated noise value to determine a final signal to noise for each ROI as described previously^50^.


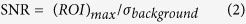


Following ROI detection described above, we limited our dataset only to ROIs with minimal overlapping areas then refined the selected ROIs based on morphology and dynamic activity by the observers blinded to trial timing or events. Each trace was normalized with the following convention:


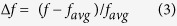


where *f*_*avg*_ is the averaged value of the trace. The Δ*f* was than rescaled so that the maximal value of the trace was equal to 100%.

#### Statistical Analysis and Quantification of Task Relevant ROI’s

Power analysis was first utilized to determine the ROI sample size necessary to identify task-mediated changes for both positive and negative responses. We chose an effect size of 20% which reflects the near minimum number of neurons reported as task-modulated in previous electrophysiology studies^23^,^26^,^27^,^34^,^35^. Power analysis was performed using G*Power 3.1.9.2 (http://www.gpower.hhu.de) utilizing an α = 0.05 and a power (1−β) value of 0.80 for the t-statistics used in this analysis^51^. This revealed a minimal sample size of 150 neurons. We exceeded this number in all three animals described here, so further statistical analysis was performed. All statistics were carried out in MATLAB using the statistics toolbox.

Behavioral analysis of task-relevant ROIs was conducted following the image stabilization and ROI extraction process. We took ROI extracted traces and aligned them by trial to identify task relevance using a paired t-test in individual subjects as described next. We compared the area under the calcium trace curve during the baseline period and the task period of each trial. The baseline period and the task period were defined as the 2 second window before and after sound onset, respectively. A ROI was determined task relevant if the p-value was less than 0.05. We further classified the task relevant ROIs based on their averaged area across all correct trials. A ROI was positively modulated if its averaged area during the task period was larger than that during the baseline period, and was negatively modulated if smaller. To compare across the population, repeated measures ANOVA was used.

To calculate spatial distributions, we performed a resampling test for individual subjects. Briefly, we tested the hypothesis that the positively modulated ROIs represented a more sparsely clustered population of neurons than the remaining ROIs. We used the median distance between ROIs as a test statistic. We resampled 500 times from the total ROI population, with replacement between resamples, to create a null hypothesis distribution on our test statistic. For each sample, we drew an equal number of ROIs as our test population without replacement from the total ROI population and calculated the test statistic. One-sided significance was determined by comparing the observed test statistic value against the distribution of the null hypothesis test statistic values. We then counted the number of null distribution values that were lower than the observed test statistic and divided by the number of resamples. To evaluate the population effects a Mann-Whitney Rank Sum was used.

## Additional Information

**How to cite this article**: Mohammed, A. I. *et al.* An integrative approach for analyzing hundreds of neurons in task performing mice using wide-field calcium imaging. *Sci. Rep.* **6**, 20986; doi: 10.1038/srep20986 (2016).

## Supplementary Material

Supplementary Information

Supplementary Video 1

Supplementary Video 2

Supplementary Video 3

## Figures and Tables

**Table 1:** Summary of ROIs detected and motion indexes from all animals in study.

Dataset	Image shift(µm/frame, mean ± sd)	# of ROI	Positively modulated	Negatively modulated
Mouse26 (selected ROIs)	1.18 ± 1.65	422	81 (19.19%)	102 (24.17)
Mouse 26 (all ROIs)	1.18 ± 1.65	1086	216 (19.89%)	374 (34.44%)
Mouse 26 (no motion correction, selected ROIs)	N/A	422	100 (23.70%)	128 (33.33%)
Mouse 26 (no motion correction, all ROIs)	N/A	1086	304 (27.99%)	330 (30.39%)
Mouse 22 (all ROIs)	1.40 ± 1.56	1797	277 (15.41%)	119 (6.62%)
Mouse 23 (all ROIs)	0.31 ± 0.75	763	471 (61.73%)	43 (5.64%)

**Table 2:** Behavioral performance and imaging signal to noise ratio.

DataSet	Correct Trials	Incorrect Trials	Signal to Noise Ratio
Mouse 26	33	7	20.38 +/- 0.68
Mouse 22	30	10	8.56 +/- 1.57
Mouse 23	36	4	6.70 +/- 0.19

**Table 3:** Spatial clustering of neurons by modulation type.

Type	Positive modulated (# of neurons/50 μm ± sd)	Negative modulated (# of neurons/50 μm ± sd)	Non modulated (# of neurons/50 μm ± sd)
Positively modulated (all)	1.17 +/- 1.25	0.41 +/- 0.90	2.01 +/- 2.22
Negatively modulated (all)	0.81 +/- 1.22	1.66 +/- 1.62	3.02 +/- 1.88
Non modulated (all)	0.81 +/- 1.03	0.62 +/- 1.08	3.33 +/- 2.32

**Table 4:** Image Processing Comparison for 30 GB data file.

Software	RAM Required	Processing Time	User Input time
Software used here	4 GB	1 hr	0.10 hrs
ImageJ (TurboReg)	80 + GB	4.5 hrs	0.50 hrs
