# Supplementary material for: An integrative approach for analyzing hundreds of neurons in task performing mice using wide-field calcium imaging
Source: Sci Rep. 2016 Feb 8;6:20986. doi: 10.1038/srep20986 (PMC4745097; doi:10.1038/srep20986)

**Supplemental Information:**

# An integrative approach for analyzing hundreds of neurons in task performing mice using wide-field calcium imaging

*Ali I. Mohammed[1], Howard J. Gritton[1], Hua-an Tseng[1], Mark E. Bucklin[1], Zhaojie Yao and Xue Han**

***Boston University, Department of Biomedical Engineering, Boston, MA 02215***

[1]*These authors contributed equally.*

**Corresponding Author.*

**Supplemental Figure 1:** (A) Non-motion corrected ROIs from the same recording session shown in Figure 3A. Bottom trace represents image registration index. Inset illustrates low amplitude physiological rhythms present in the calcium signal likely due to micro movements. (B) Same ROIs as shown above in figure S1A following motion correction. Note that physiological artifacts are removed. Large amplitude motion is largely attenuated, although in some instances remnants of the motion remain as small deflections within the ROI signal.

**Supplemental Figure 2:** Wide field ROI maps for other study subjects. Maps represent all ROIs automatically generated by the algorithm for the other mice analyzed and compare to the map shown in Figure 2A.

**Supplemental Figure 3:** Histograms of distance between cells that are positively modulated (A) and non-modulated (B), as defined in Fig. 5.

**Supplemental Figure 4:** Flow chart highlighting image processing steps used within this paper. Individual functions and their resulting outputs from each step are described in the downloadable user manual.

**Supplemental Video 1:** Full imaging window throughout a single trial session in real time. Video represents 15 seconds of recording, beginning 5 seconds prior to cue and extending until 10 seconds after cue ends. Top left corner is annotated with trial information. S=Sound, D=Delay, P=Puff.

**Supplemental Video 2:** Magnified 201x201 pixel area from the image shown in Supplemental Video 1. The image is the centered portion of the entire recording field and is annotated with trial information. S=Sound, D=Delay, P=Puff.

**Supplemental Video 3:** Magnified 201x201 pixel region shown with ROI 266 from figure 3B in the center and outlined with a black circle. Note ROI 266 shows spontaneous activity in the

inter-trial interval, but shows significantly little activity across all trials during the sound or puff interval as described in the manuscript.

# SFigure 1

A

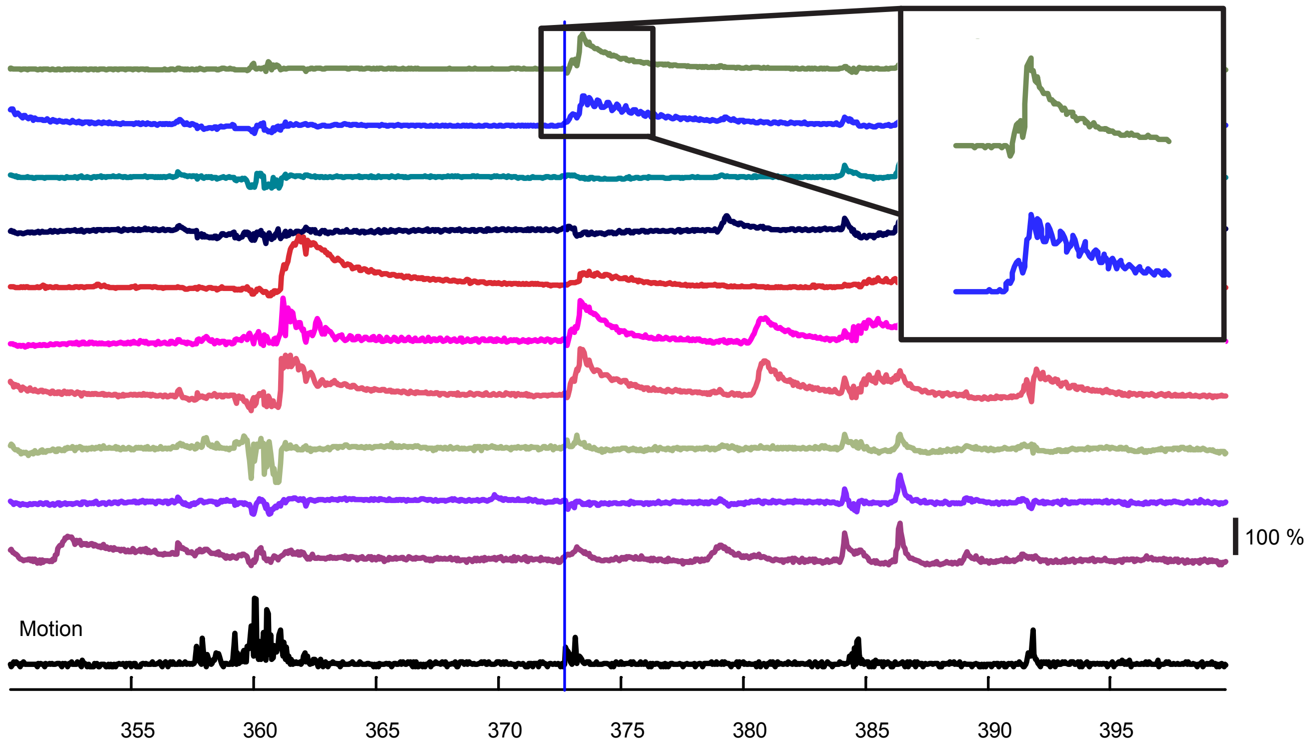
100 %
Motion
355
360
365
370
375
380
385
390
395

B

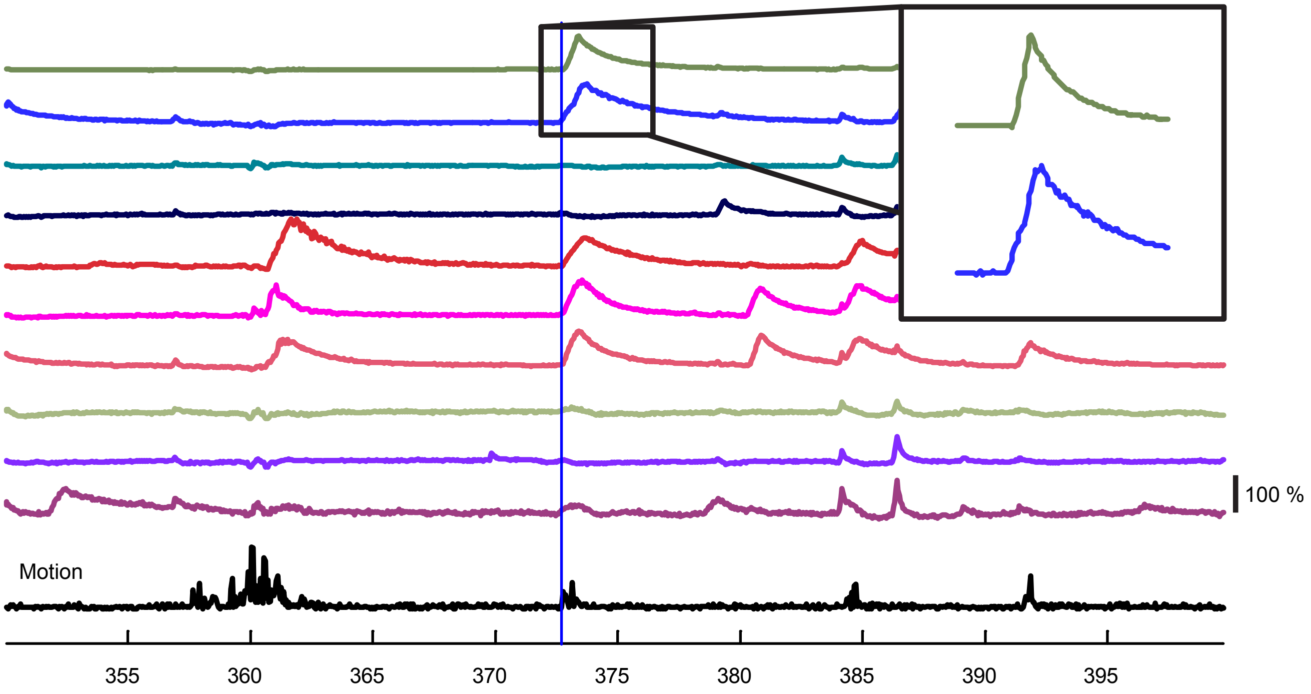
100 %
Motion
355
360
365
370
375
380
385
390
395

# SFigure 2

Mouse 22

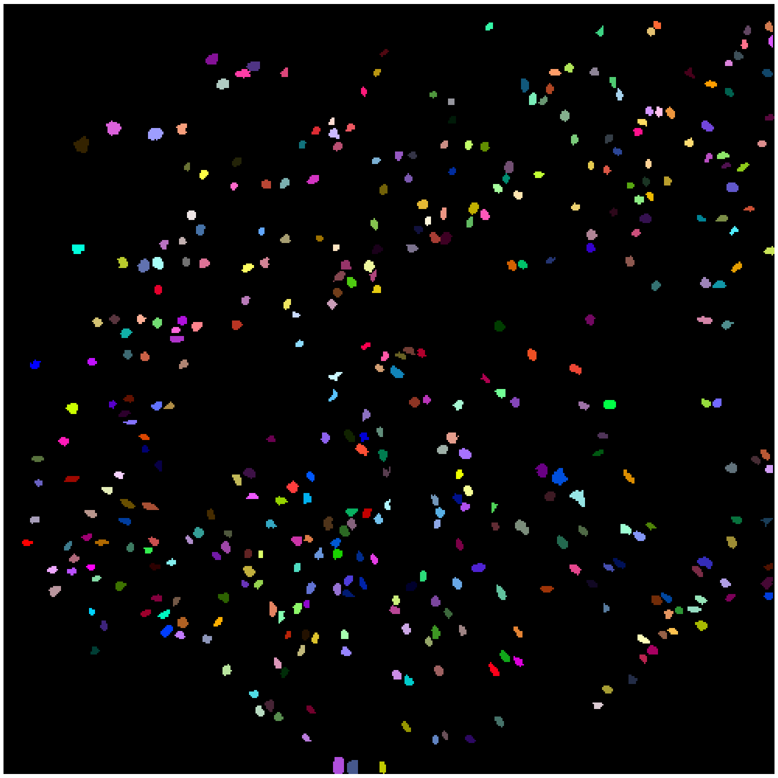

Mouse 23

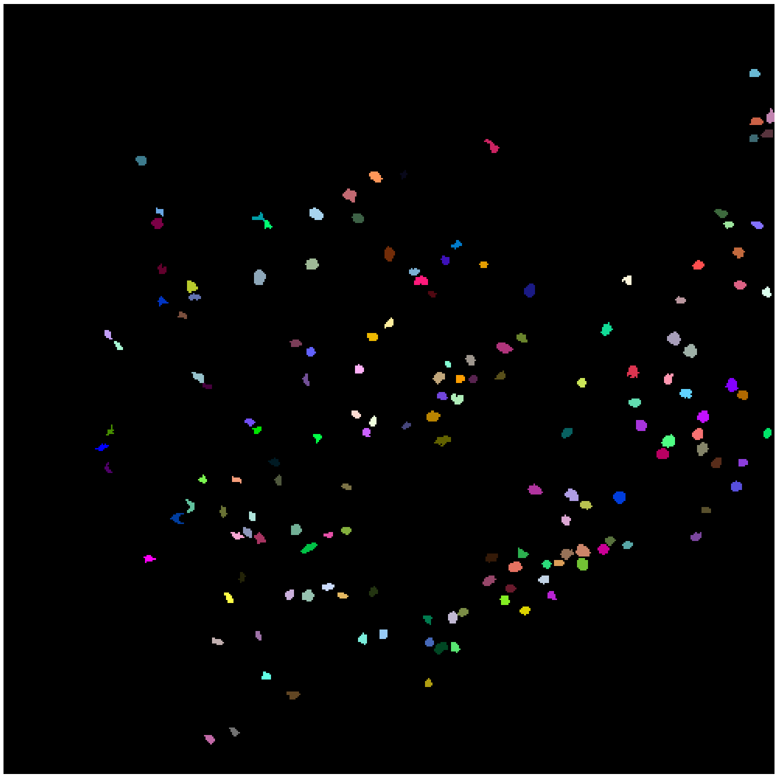

SFigure 3

A

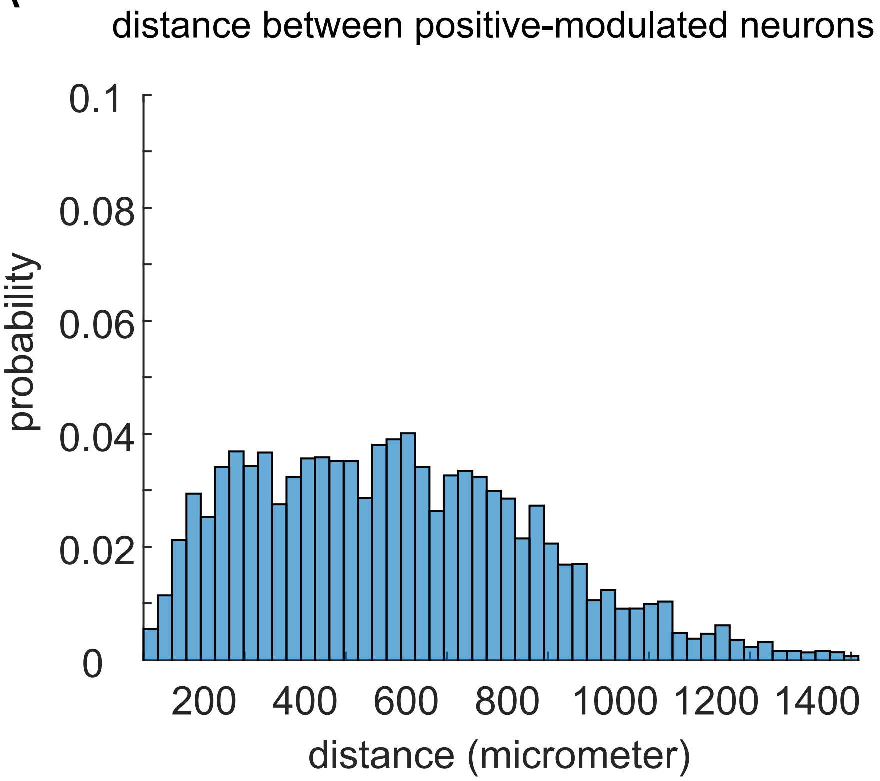
distance between positive-modulated neurons
0.1
0.08
0.06
0.04
0.02
0
probability
200
400
600
800
1000
1200
1400
distance (micrometer)

B

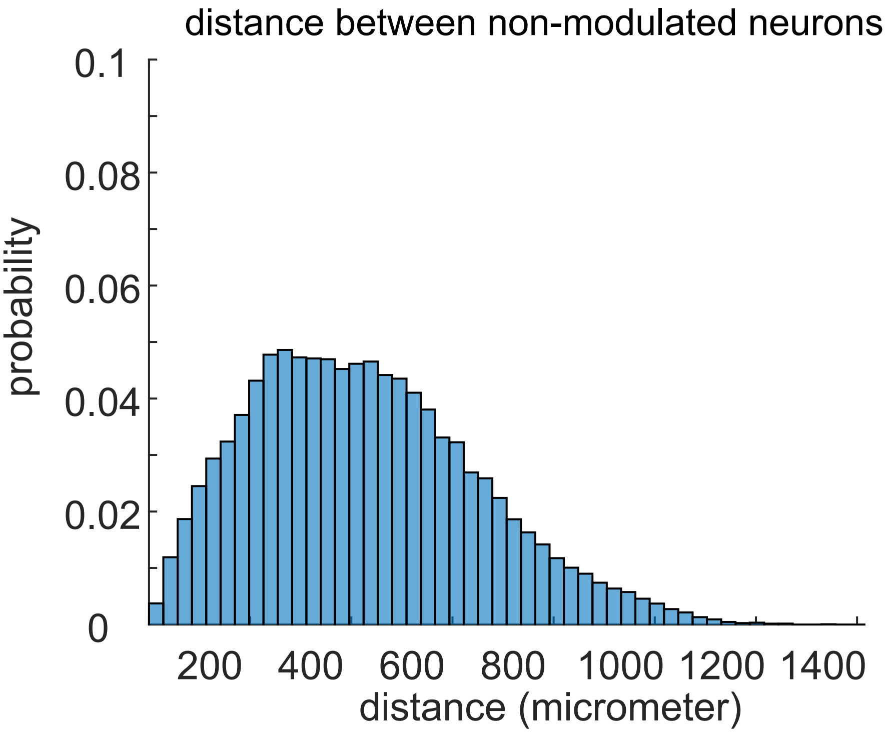
distance between non-modulated neurons
0.1
0.08
0.06
0.04
0.02
0
probability
200
400
600
800
1000
1200
1400
distance (micrometer)

SFigure 4

# Software: Organization

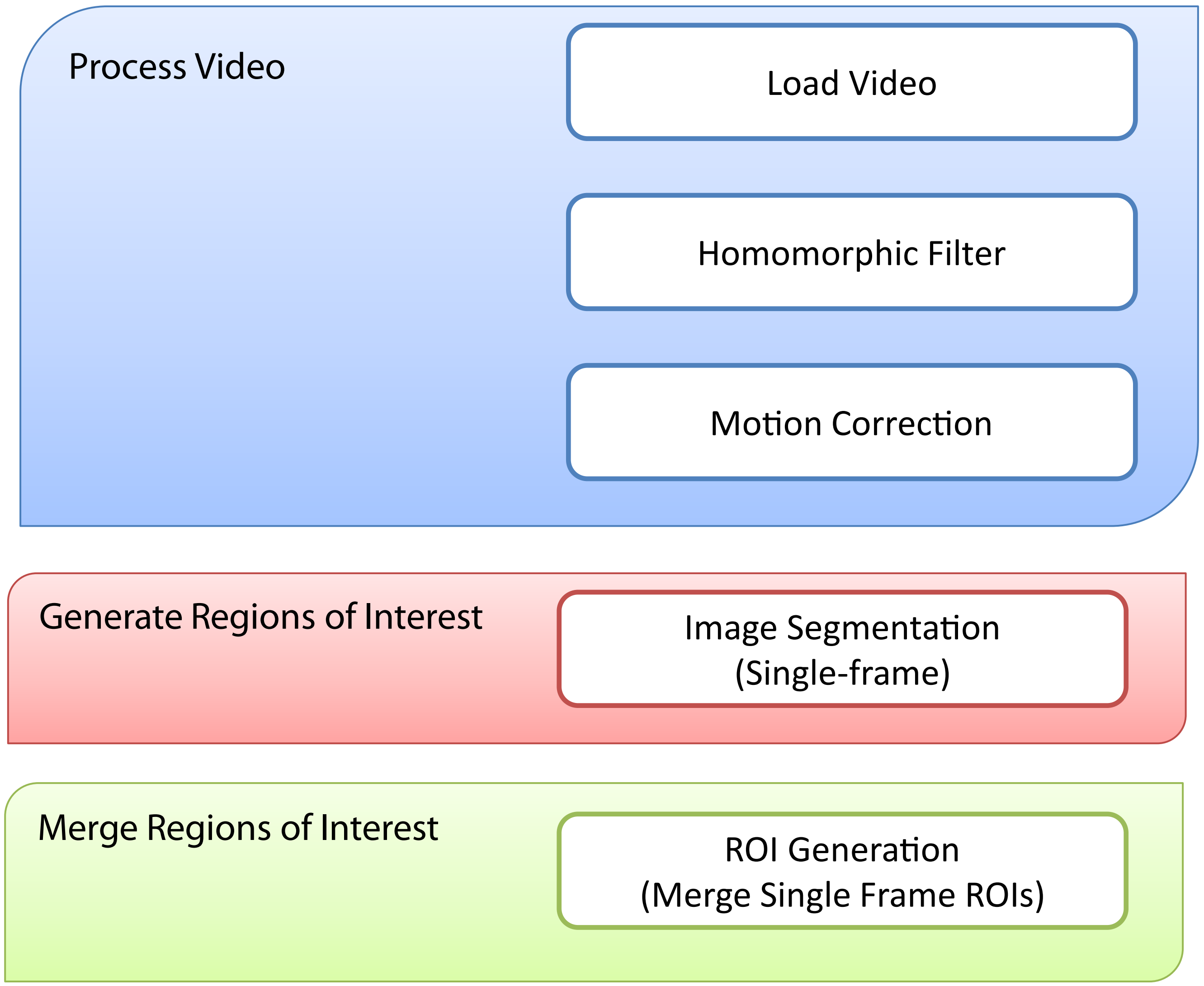

Supplement: Supplementary Information [file srep20986-s1.pdf]
